# Effectiveness of Pharmacological Agents and Validation of Diagnostic Scales for the Management of Paroxysmal Sympathetic Hyperactivity in Hispanics

**DOI:** 10.3389/fneur.2020.603011

**Published:** 2020-11-16

**Authors:** Alaa K. Abdelhakiem, Annelyn Torres-Reveron, Juan M. Padilla

**Affiliations:** ^1^DHR Health Pharmacy Residency Program, Edinburg, TX, United States; ^2^Texas A&M Irma Lerma Rangel College of Pharmacy, Kingsville, TX, United States; ^3^DHR Health Institute for Research and Development, Edinburg, TX, United States; ^4^DHR Health Neuroscience Institute, Edinburg, TX, United States

**Keywords:** autonomic nervous system, traumatic brain injury, tachycardia, hypertension, tachypnea, hyperthermia

## Abstract

The identification and treatment of paroxysmal sympathetic hyperactivity (PSH) still present a significant challenge. We assessed the efficacy of pharmacological agents in treating PSH symptoms and the validity of the diagnostic scales in a cohort of Hispanic patients. A retrospective chart review of cases from a single hospital was conducted in 464 records. Exclusion criteria included underlying conditions such as severe infection. Only nine patients remained in the cohort after examining their clinical records, corresponding to the following diagnoses: traumatic brain injury, subdural hemorrhage, anoxic or ischemic encephalopathy, pneumocephalus, and cerebral palsy. Using the PSH likelihood scale, six of the nine patients were identified with a score of 17 or higher, corresponding to a “probable” PSH, and three patients obtained a score between 8 and 16, corresponding to a “possible” PSH diagnosis. The top three classes of medications used were beta-blockers, antipyretics, and opioids. Benzodiazepines and neuromodulators were also frequently used in patients with trauma, but not in the ones with non-traumatic injuries. Interestingly, 75% of the patients have prescribed levothyroxine as a home medication after the PSH presentation. Medication administration did not follow a specific pattern, suggesting high variability in the management of PSH within our setting, requiring further research. Our results suggest that the pituitary axis might be involved in the progression of PSH. Establishing a specific medical code (e.g., ICD-10) describing PSH as a single entity is essential for appropriate identification and management.

## Introduction

Excessive sympathetic nervous system activity can develop after severe acquired brain injury. The sympathetic discharge can have a striking presentation with paroxysmal tachycardia, arterial hypertension, tachypnea, hyperthermia, and decerebrate posturing occurring in response to afferent stimulation (1). Clinical features resembling paroxysmal sympathetic hyperactivity (PSH) were first described after traumatic brain injury (TBI) by Wilder Penfield (2). Since Penfield's initial description, the same syndrome has had over 31 different labels. Some of these labels were descriptive (e.g., dysautonomia, autonomic storms, or sympathetic storms), some referred to an assumed epileptic mechanism (e.g., autonomic seizures), and some to the site of damage [e.g., hypothalamic storms; (3–5)]. The current consensus is that autonomic hyperactivity in PSH concerns only the sympathetic nervous system (1, 6, 7). The absence of a clear definition or terminology for PSH was probably a consequence of its under-recognition, despite its relatively high incidence after severe brain damage (8, 9), the well-recognized association with morbidity (1, 10), and increased health- care and societal costs (11).

Several classes of drugs have been used to treat patients with PSH (12), with varying success. The syndrome is likely to be mechanistically heterogeneous, and identification of the dominant pathophysiological processes responsible for the clinical picture in individual patients could allow more rational matching of patients to therapies and move toward precision-medicine approaches in the treatment of PSH. The recent development of diagnostic criteria for the condition has provided the essential first step for such an exercise since these criteria can be used to clearly define an initial population of patients for such therapeutic stratification. The objective of the study was to understand whether the current pharmacological agents used to treat PSH symptoms are effective in controlling PSH symptoms, specifically in a population of predominantly Hispanic patients. We hypothesized that the current treatment modalities were not effective in resolving the symptoms for PSH. Understanding how pharmacological agents are used in light of the variety of symptoms will help improve the diagnosis and management of the disease.

## Methods

### Study Design

This study is a retrospective chart review of cases only, on patients admitted from 08/31/2014, to 02/29/2020 from a single academic medical facility. Patients' charts were included for consideration for the following diagnoses: (1) traumatic brain injury with and without loss of consciousness of unspecified duration; (2) anoxic brain injury; (3) severe sepsis without septic shock; (4) systemic inflammatory response syndrome (SIRS) of non-infectious origin without acute organ dysfunction; (5) SIRS of non-infectious origin with acute organ dysfunction; (6) hypoxic-ischemic encephalopathy (HIE); (7) encephalopathy with at least two symptomologies: tachycardia, hypertension, tachypnea, fever, diaphoresis, and posturing. Patients were followed longitudinally from the identified onset of injury, to discharge from the hospital, or eventual death: this included records from the emergency room, intensive care unit, step-down unit, and regular floor. Records from rehabilitation facilities, were not considered for this study. Patients of all ages and both sexes were considered for inclusion. A randomly computer-generated alphabetical code was assigned to each patient before full data extraction began.

A patient chart was excluded from the full review if any of the following diagnoses were documented in the record: hyperthyroidism, pulmonary embolism, substance withdrawal, serotonin syndrome, sepsis, pheochromocytoma, neuroleptic malignant syndrome, or malignant hyperthermia.

### Variables

To reach a possible diagnosis of PSH, the following criteria were used (5): (1) fever, defined as a body temperature >38.3°C, (2) tachycardia, defined as heart rate >120 beats/min or >100 beats/min if the patient was being treated with a beta-blocker, (3) hypertension defined as systolic blood pressure >160 mmHg or >140 mmHg if the patient was on antihypertensives, (4) tachypnea, defined as a respiratory rate >25 breaths/min, (5) diaphoresis, (6) presence of posturing (change in posturing or spontaneous posturing), and (7) rigidity or spasticity. There must have been more than one episode daily from at least four of the criteria described above.

### Data Source and Measurements

Investigators requested a report from the hospital listing the clinical encounters that met the diagnosis inclusion criteria using the corresponding International Classification of Disease 10th Revision (ICD10) codes for each criterion. A single reviewer (AKA) applied the exclusion criteria to generate a list of possible patients with PSH. Each record was then analyzed in detail to search for doctor's notes that would fall within the exclusion criteria, but that might not have been explicitly coded in the chart. This secondary review was verified by the principal investigator (JMP), further eliminating additional patients. We applied the previously published scales (13) for the Severity of Clinical Features Assessment Tool (SCFAT) and the Diagnosis Likelihood Tool (DLT), classifying each patient as “probable,” “possible” or “unlikely,” depending on the total sum of both scales. We used the data from the hospitalization period immediately following the documented condition (traumatic brain injury, stroke, etc.) and using the highest values of the clinical features reached during a single episode during a given day. The SCFAT was calculated for at least four different days to validate the scale within our study population. For pediatric patients, the values were adjusted for the specific normal values within the age group at the time of the observed PSH episodes as previously reported (12, 14). Calculations of the SCFAT and the DLT were done by one investigator (ATR) and verified by the principal investigator (JMP).

### Reduction of Bias

During the methodological design of the study, some of the observed ICD10 codes used for documenting the possible presence of PSH were G90.8 and G90.9 (a disorder of the autonomic nervous system), though, infrequently used. One of the main methodological challenges was that an explicit diagnosis of PSH was not present in the patient's charts (except for one patient). If the list of symptoms in a particular patient could have been explained by any other cause (e.g., triggered exclusively by infection) the patient was excluded. The selection of patients was not based on outcomes, but solely by the presence of symptoms. Since the medications given to the patient was one of the outcomes, this criterion was excluded from the selection process and only collected after inclusion and exclusion criteria were met. Data collection was done by the same investigator and verified by the principal investigator.

### Quantitative Variables

The following parameters were extracted: length of stay, heart rate, respiratory rate, blood pressure, temperature, Glasgow Coma Scale (GCS), intracranial pressure (IP), reactivity to pain, the presence of posturing, rigidity, spasticity, and diaphoresis. Other variables included the number of episodes per day, number of days presenting episodes, mode of injury, all medications given during the hospitalization as well as all medications given during discharge. The PSH diagnosis likelihood score is a combined propensity value resulting from the sum of the SCFAT and the DLT. The maximum score a patient could obtain in the SCFAT is 18 points based on a score of 0–3, for heart rate, respiratory rate, systolic blood pressure, temperature, sweating, and posturing during episodes. The maximum score a patient could obtain for the DLT is 11 based on a binary score of 0 or 1 (presence or absence) of the following: clinical features occur simultaneously, episodes are paroxysmal in nature, sympathetic over-reactivity to normal non-painful stimuli, features persist for 3 consecutive days, features persist for 2 weeks post brain injury, features persist despite treatment of alternative differential diagnosis, medication is administered to decrease symptoms, two or more episodes daily, absence of parasympathetic features during episodes, absence of other presumed causes or features, and antecedent acquired brain injury. The maximum total points a patient could obtain from the combined scales is 29. Based on this score, PSH is “unlikely” if the patient has <8 points, “possible” if the score falls between 8 and 16 and “probable” if the score is 17 points or higher.

### Statistical Methods

Values for each patient were quantified individually. In the appropriate instances, we used descriptive statistics, which included sum, averages, and standard deviations. A Fisher exact test was used to compare the frequency of medications used during the inpatient period and after discharge. Statistical significance was set at *p* <0.05 and analyses were done using JMP-Statistical software (Version 15, SAS, Cary, NC, United States). Data was collected de-identified using Microsoft Excel, and final figures for publication were prepared using GraphPad Prism (San Diego, CA, United States).

## Results

### Patients Demographics and Other Characteristics

The initial report obtained from the hospital included 464 encounters that fit the diagnostic criteria for the presence of four or more of the symptoms related to PSH, after applying exclusion criteria for other presumed causes of the symptoms, only 24 encounters belonging to nine patients remained: seven adults and two pediatric. Table 1 describes the demographic information of the patients as well as the length of stay (LOS) and their initial admitting diagnosis, as reported in the medical record for the period during which the PSH symptoms were observed, the GCS upon admission, location of brain injury, location of the patient, and last known outcome. The average age of the patients was 38.6 ± 21.7 years old, and their average length of stay was 22.2 ± 19.5 days. Three out of the nine patients were females, and also three out of the nine had traumatic brain injuries. All of our patient's self-reported ethnicity was Hispanic/Latino, which is typical for our geographical region. The average GCS upon admission was 6.57 ± 3.55 points. Only three patients are confirmed to be alive, while five were confirmed dead from the medical record. One patient last known information was from 2015, and it was not clear whether he was still alive.

**Table 1:** Patients demographic and baseline clinical characteristics.

**Patient ID**	**Age**	**Gender**	**Ethnicity**	**LOS**	**GCS**	**Reason for admitting**	Brain location^*^	**Setting**	**Last known status**
PCIOKGE	17	M	Hispanic/Latino	42	3	MVA, subdural hemorrhage	Diffuse axonal injury, sheer injury corona radiata, CC, midbrain	ER, ICU	Alive, uses aid to ambulate
PCKCKEJ	49	M	Hispanic/Latino	12	12	Seizures, Fever	Cortical atrophy, history of anoxic brain injury	ICU	Quadriplegic as of May, 2015.
PGYWAUC	65	M	Hispanic/Latino	23	8	Acute encephalopathy	Third ventricle and diencephalon	ER, ICU	Expired outside of hospital.
PJDABTB	5	F	Hispanic/Latino	9.5^#^	N/A	Seizure disorder	Microcephaly, basal ganglia and diencephalon	ICU	Alive, vegetative state
PKDTGHT	48	M	Hispanic/Latino	45	4	Gunshot wound	Thalamus and basal ganglia/diencephalon	ER, ICU	Expired several months after discharge
POKNYTV	30	M	Hispanic/Latino	53	6	Traumatic intraventricular hemorrhage	Diffuse axonal injury, corona radiata, CC, midbrain, diencephalon	ER, ICU	Alive. Spastic hemispheric atrophy
PSFLWFK	40	F	Hispanic/Latino	6^∧^	N/A	Anoxic encephalopathy and brain death	Global ischemic, anoxic injury	ER, ICU	Expired in hospital
PTQYMNX	24	F	Hispanic/Latino	15^∞^	10	Changes in mental status, comatose	Periventricular white matter and basal ganglia	ER, ICU	Expired outside hospital
PUQXAUX	70	M	Hispanic/Latino	4	3	Ischemic stroke	Head of the caudate nucleus and left genu of the CC	ER, ICU	Expired at hospice care

*LOS, length of stay in days; ER, emergency room; ICU, intensive care unit; CC, corpus callosum*.

* *Based on the first brain CT or MRI available in the medical record after injury onset*.

# *Average LOS based on multiple ER visits and hospitalizations since birth*.

∧ *Patient had 340 ER encounters and 6 ICU stays from 2013 to 2016 complaining of shortness of breath*.

∞ *Mother reports fever onset of unknown etiology for 6 continuous months*.

### Application of Diagnostic Scales

The SCFAT calculations are shown in Table 2. The average SCFAT score for all the patients was 10.5 ± 3.6 points. Two of the patients presented all six of the clinical features, and both of these patients had traumatic injuries. Severe increases in respiratory rate (≥30 breaths per min.) and in systolic blood pressure (≥180 mmHg) were the clinical symptoms most frequently observed, followed by the presence of posturing and a severely increased heart rate (≥140 beats per min.). The assessment of the DLS is presented in Table 3. Three of the patients showed a maximum score of 11 points. All of the patients in the cohort had an antecedent of acquired brain injury, and also all of them received some type of medication to decrease the symptoms. The other most frequent features observed were the simultaneity of features, the persistence of symptoms for more than 3 consecutive days, and more than two episodes daily. Six of the nine patients had a total score for the probability of PSH of 17 or higher (Table 4). The average score for the patients who showed a “probable” classification was 22 points. For the remaining three patients who scored below 17 points, the average score was 12 points. This puts them under the classification of “possible.” None of our patients were categorized as “unlikely” given that our exclusion criteria were designed to reject those patients for which the measures for PSH were not met.

**Table 2:** Severity of clinical features assessment (SCFA) for each patient.

**Patient ID**	**PCIOKGE**	**PCKCKEJ**	**PGYWAUC**	**PJDABTB**	**PKDTGHT**	**POKNYTV**	**PSFLWFK**	**PTQYMNX**	**PUQXAUX**
Heart Rate	3	1	3	0	2	1	3	2	2
Respiratory Rate	1	1	3	3	3	3	3	1	2
Systolic BP	2	2	3	1	3	3	3	1	2
Temperature	2	0	3	1	1	0	3	1	1
Sweating	1	0	0	1	3	0	0	0	0
Posturing	3	1	2	1	3	3	1	3	0
Total	12	5	14	7	15	10	13	8	7

*0 = none, 1 = mild, 2 = moderate, 3 = severe. See (5) for additional information*.

**Table 3:** Diagnosis Likelihood Tool (DLT) for PSH.

**Patient ID**	**PCIOKGE**	**PCKCKEJ**	**PGYWAUC**	**PJDABTB**	**PKDTGHT**	**POKNYTV**	**PSFLWFK**	**PTQYMNX**	**PUQXAUX**
Clinical features occur simultaneously	1	0	1	1	1	1	1	1	1
Episodes are paroxysmal	1	0	1	0	1	1	1	1	1
Over reactivity to non-painful stimuli	1	0	0	0	1	1	0	1	0
Persist for 3 consecutive days or more	1	0	1	1	1	1	1	1	1
Persist for more than 2 wks. post brain injury	1	1	1	1	1	1	0	1	0
Persist despite treatment or differential diagnosis	1	0	1	0	1	1	0	1	0
Medication given to decrease sympathetic features	1	1	1	1	1	1	1	1	1
Two or more episodes daily	1	0	1	1	1	1	1	1	1
No parasympathetic features during episodes	1	0	0	0	1	1	1	1	0
No other presumed causes or features	1	1	1	0	1	1	0	1	1
Antecedent of acquired brain injury	1	1	1	1	1	1	1	1	1
Total	11	4	9	6	11	11	7	11	7

**Table 4:** Summary and assessment of PSH using the results from Tables 2, 3.

**Patient ID**	**PCIOKGE**	**PCKCKEJ**	**PGYWAUC**	**PJDABTB**	**PKDTGHT**	**POKNYTV**	**PSFLWFK**	**PTQYMNX**	**PUQXAUX**
CFA	12	5	14	7	15	10	13	8	7
DLT	11	4	9	6	10	11	7	11	7
Sum	23	9	23	13	25	21	20	19	14
PSH	PROBABLE	POSSIBLE	PROBABLE	POSSIBLE	PROBABLE	PROBABLE	PROBABLE	PROBABLE	POSSIBLE
Final Dx	Trauma	Anoxic encephalo-pathy	Pneumoce-phalus	Ischemic encephalo-pathy	Trauma	Trauma	Anoxic brain damage	Cerebral Palsy	Ischemic brain disease

*Criteria for PSH Diagnostic Likelihood: <8, Unlikely; 8–16, Possible; >17 Probable. Dx, diagnosis*.

### Medication Effectiveness Assessment

**Figure 1:**
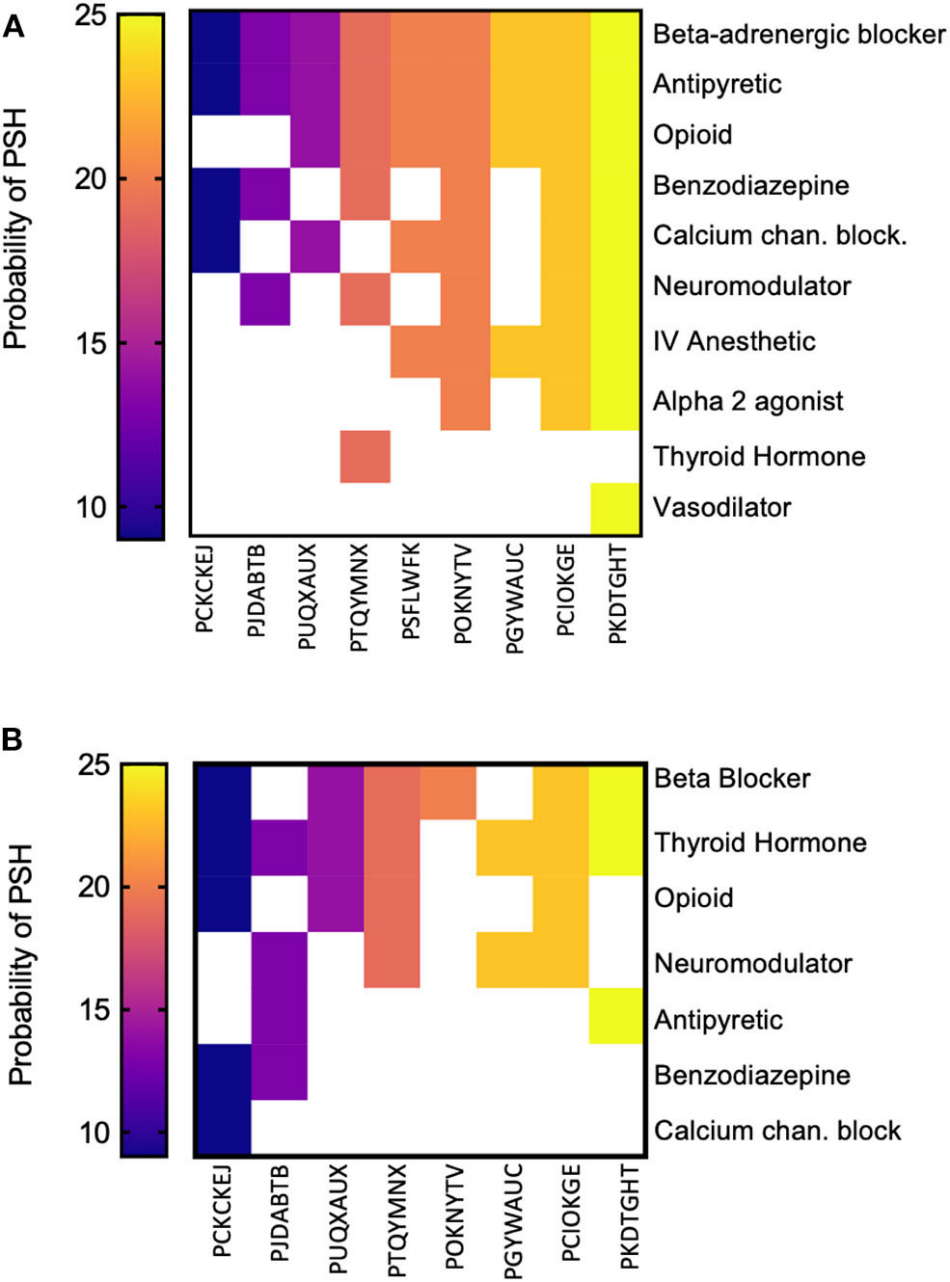
Medication given during the inpatient period and post-discharge. **(A)** The probability of PSH, as calculated for each patient, was ordered in magnitude (left to right) against the most frequent to least frequently used medications (top to bottom) during the inpatient period. **(B)** Upon discharge, the classes of medications used by the patients was quantified in the same manner as in **(A)**. Only eight patients are illustrated since one patient died at the hospital.

The frequency of medications given during the inpatient period is depicted in Figure 1. Medication classes given during the inpatient period were graphed against the PSH likelihood score. We found that all patients received beta-blockers, which is the first line of treatment for tachycardia. Antipyretics to reduce hyperthermia and opioids to treat pain were the second and third most used medications in the patient cohort, respectively. It is also noticeable that the top five probable PSH patients (trauma and non-trauma) all received beta-blockers, opioids, antipyretics, and IV anesthetics. In addition, all three trauma patients also received the alpha 2 agonists, neuromodulators, calcium channel blockers, and benzodiazepines. The use of other types of medications varied depending on the patient and did not correspond to the severity of the PSH. The number and classes of medications that were maintained for in-home use are shown in Figure 1B. Data for only eight out of the nine patients are presented, given that one patient died at the hospital (PSFLWFK). Not all patients maintained the beta-blocker, antipyretic, or the opioid upon discharge, suggesting that symptoms partially or totally resolved. Of striking relevance is the observation that only one patient was using thyroxine before the brain insult event, but after hospitalization, six out of eight patients (75%) were prescribed thyroxine. In only one patient (PCIOKGE), thyroxine was not prescribed during inpatient or post-discharge medication. Fisher exact test revealed that there was a significant change in the number of patients using thyroxine during the at-home medications period vs. the inpatient period (*p* = 0.003). Based on the current cohort of patients, the relative risk of being prescribed thyroxine after a “possible” or “probable” diagnosis of PSH was 8.0 (95% CI: 1.93, 45.09). Upon a further review of the chart, we observed that the initiation of thyroid medication ranged from 1 to 12 months. Thyroid medication use was not associated with age (*X*^2^ = 2.77, d.f. = 1, *p* > 0.05) or site of brain injury (*X*^2^ = 3.73, d.f. = 4, *p* > 0.05).

We looked at the specific medications given during the inpatient period for the six patients that scored 17 or higher in the PSH likelihood scale subdivided by “non-traumatic” (Table 5) and “traumatic” (Table 6), based on their cause of injury. The PSH likelihood scale criteria served as the guiding template to assess medication response, meaning, we ranked the response as positive if the patient no longer met the threshold for specific criterion. Non-traumatic patients received fewer classes of medications as compared to traumatic patients. Specifically, alpha 2 agonist was not administered. Interestingly, the IV anesthetic propofol produced a positive response in managing the refractory symptoms in both trauma and non-trauma patients (3 out of 5 patients).

**Table 5:** List of medications and response assessment for patients with non-traumatic PSH with a classification of “Probable” from Table 4.

**Drug classes**	**Medication response**			
**Opioids**	**Hypertension**	**Tachycardia**	**Pain**	**Respiratory rate**
Fentanyl	+	+	0	–
Morphine	+	–	++	–
**IV anesthetics**	**Hypertension**	**Tachycardia**	**Respiratory rate**	**Fever**
Propofol	+	+	–	–
**Beta-adrenergic blockers**	**Hypertension**	**Tachycardia**	**Diaphoresis**	**Dystonia**
Labetalol	+	+	0	0
Metoprolol	-	+	0	0
**Neuromodulators**	**Fever**	**Diaphoresis**	**Pain**	**Spasticity**
Baclofen	+	0	+	+
**Benzodiazepines**	**Hypertension**	**Tachycardia**	**Respiratory rate**	
Diazepam	+	+	–	
**Calcium channel blockers**	**Hypertension**	**Tachycardia**		
Amlodipine	+	–		
**Antipyretic**	**Fever**	**Pain**		
Acetaminophen	+++	0		

*Hypertension: systolic blood pressure >160 mmHg or >140 mmHg if on antihypertensive; Tachycardia: HR >120 bpm or >100 bpm if on beta blocker; Fever: >38.3°C. Tachypnea: respiratory rate >25*.

**Table 6:** List of medications and response assessment for patients with traumatic PSH with a classification of “Probable” from Table 4.

**Drug classes**	**Medication response**			
**Opioids**	**Hypertension**	**Tachycardia**	**Pain**	**Respiratory Rate**
Fentanyl	+++	++	0	++
Morphine	+	+	0	–
Meperidine	+	–	0	+
**IV anesthetics**	**Hypertension**	**Tachycardia**	**Respiratory rate**	**Fever**
Propofol	++	++	++	–
**Beta-adrenergic Blockers**	**Hypertension**	**Tachycardia**	**Diaphoresis**	**Dystonia**
Labetalol	+++	++	0	0
Metoprolol	++	+	0	0
Propranolol	+	–	0	0
**Alpha-2-agonists**	**Hypertension**	**Tachycardia**	**Pain**	**Dystonia**
Dexmedetomidine	+	+	0	0
**Neuromodulators**	**Fever**	**Diaphoresis**	**Pain**	**Spasticity**
Cyclobenzaprine	–	0	+	+
Gabapentin	–	0	0	+
Bromocriptine	–	0	0	+
Baclofen	–	0	0	+
**Benzodiazepines**	**Hypertension**	**Tachycardia**	**Respiratory rate**	
Midazolam	+++	+	–	
Lorazepam	++	+	+	
Clonazepam	+	+	+	
**Calcium channel Blockers**	**Hypertension**	**Tachycardia**		
Nicardipine	+++	–		
**Vasodilator**	**Hypertension**	**Tachycardia**		
Hydralazine	–	–		
**Antipyretic**	**Fever**	**Pain**		
Acetaminophen	++	0		

*Hypertension: systolic blood pressure >160 mmHg or >140 mmHg if on antihypertensive; Tachycardia: HR >120 bpm or >100 bpm if on beta blocker; Fever: >38.3°C*.

In patients with traumatic injuries, nine classes of medications were given to control PSH-related symptoms. The effectiveness of the medications varied both by the patient and by specific medications within the same class. From the opioids class, fentanyl was effective in controlling hypertension, tachycardia, and tachypnea. Beta-blockers controlled hypertension and tachycardia, with labetalol having an overall best response. Neuromodulators, in general, controlled spasticity, but we could not determine any preference for a particular agent as patients received different drugs within the same class. Lorazepam and clonazepam had a wider spectrum of symptom control (Table 6), but compared to midazolam, the latter was good only for controlling hypertension. Acute control of hypertension was achieved by nicardipine.

**Figure 2:**
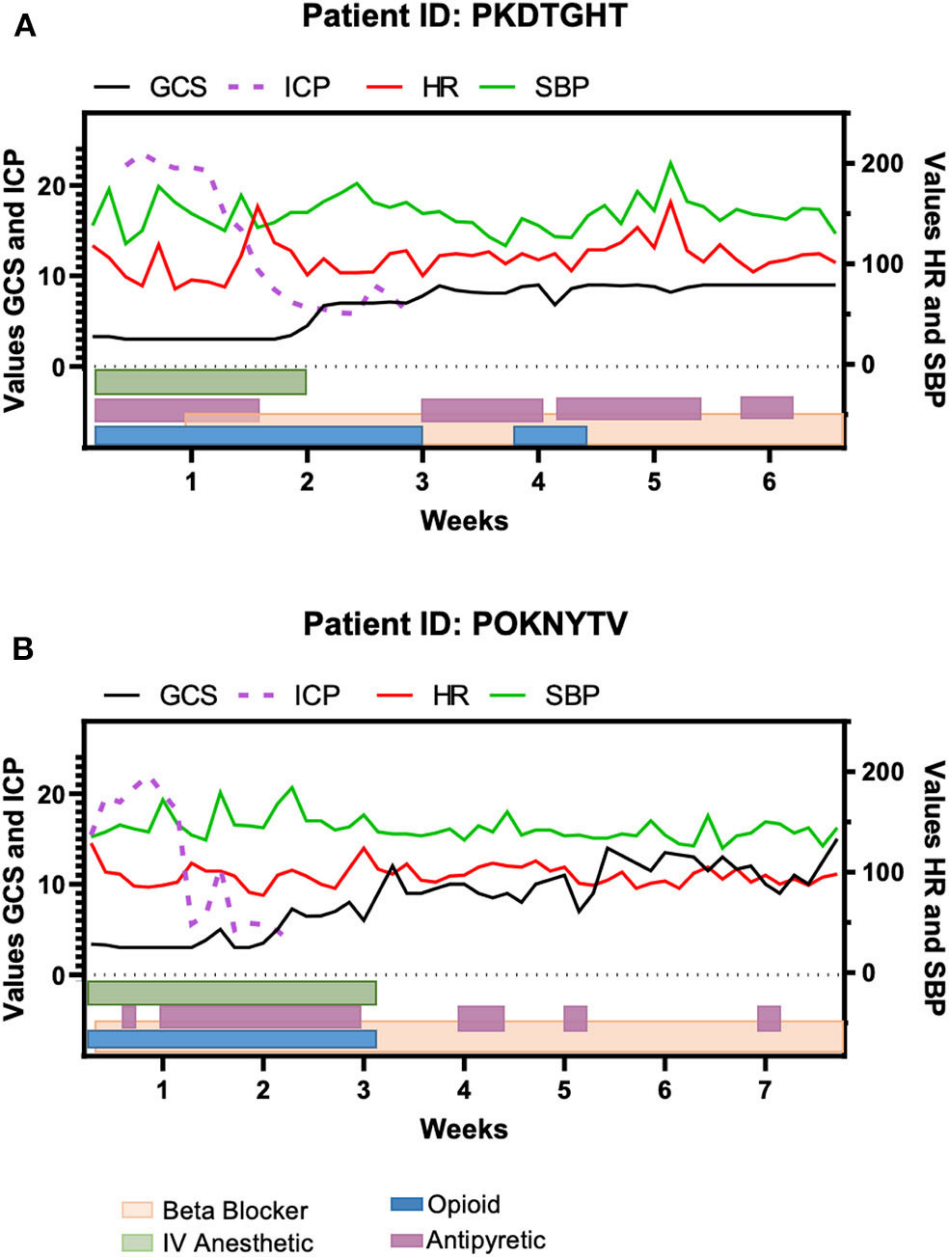
Timeline of clinical features for two of the traumatic brain injury patients and the medications given during their inpatient period. **(A)** Patient PKDTGHT was hospitalized for approximately seven weeks. Elevations in heart rate (HR) and systolic blood pressure (SBP) were still evident around week 5, despite the maintenance of medications. **(B)** Patient POKNYTV had a similar course as the patient depicted in **(A)**, but his elevation in BP and HR were less noticeable. In both patients, the Glasgow Coma Scale (GCS) improvement corresponds to the regularization of intracranial pressure (ICP).

To further explore the effectiveness of the medications used in patients with traumatic injuries, we examined the progression of PSH utilizing the heart rate (HR) and the systolic blood pressure (SBP) in parallel to the GCS and ICP during the patient's course within the Neuro-intensive care unit (Figure 2). These two patients were selected due to the similarities in injuries, time spent hospitalized, and the presentation of symptoms. While GCS and ICP are not divulged in the literature as parameters to monitor PSH, in both patients, we observed an improvement in GCS when the ICP was decreased to normal, and medications relatively controlled the spiking in HR and SBP. For patient PKDTGHT the period between weeks 3 and 4 and for patient POKNYTV the period between 3.5 and 4.5 weeks after injury were evidenced as a “quiet time” during which the spikes in HR and SBP were of lesser magnitude compared to the 1st and 2nd weeks.

## Discussion

We report a cohort of Hispanic patients with traumatic and non-traumatic brain injuries that developed PSH. The patients presented here ranged from five (5) to seventy (70) years old. Similar to what has been recently reported in the literature (12, 15), the effectiveness of pharmacological agents to treat PSH symptoms was heterogeneous across patients. Additionally, the number of medications used did not correlate to the severity of PSH, as contrasted against the PSH likelihood score. We were able to identify specific classes of medications that, when given concurrently, result in adequate control of PSH symptomatology (see Clinical Recommendations section). One of the most salient findings is that six out of the eight patients discharged required *de novo* thyroid hormone replacement, suggesting the development of hypothyroidism subsequent to the injury. While we cannot identify from the retrospective record evaluation the exact date(s) during which the hypothyroidism developed, this finding highlights the vulnerability of the pituitary axis following brain injury.

To the best of our knowledge, delayed hypothyroidism has never been reported in a cohort of patients with PSH. It has been described that immediately following the traumatic event, there is a surge in catecholamines, but the thyroid axis remained stable (16). One possible explanation arises from the anatomical location of the pituitary. The pituitary is located in the *sella turcica*, and the only connection to the hypothalamus is through the pituitary stalk. Previous investigators have suggested that the trauma could impact pituitary hormones more than the non-pituitary ones due to the damage to the stalk (17, 18). Evidence for this is reported for shearing injuries, which could result in hypothalamic-pituitary damage (19). Besides trauma, in the hypothalamus, there is a population of cells that are sensitive to hypoxic events (20, 21). Therefore, patients developing hypoxia might be an increased risk of developing hypothyroidism (17, 22). While the significant finding regarding the risk of receiving thyroid hormone replacement after a brain insult that leads to PSH was incidental, our finding pinpoint associated clinical features that require clinical management in multiple aspects, including treatment, and monitoring following brain injuries. We recognize the need for additional research in this area, given the small size of the current cohort, but it sets a clear pathway in the design of prospective studies.

We postulate that for patients with severe brain trauma, upon arrival to the hospital emergency room, a baseline panel of pituitary hormones should be collected. At a minimum, it should include thyroid-stimulating hormone, adrenocorticotropic hormone, cortisol, and prolactin. This panel will provide clinicians with the patient's baseline prior to the accident, given that pituitary hormonal changes, including TSH, could take several weeks or months to reflect a change (23, 24). Central anesthetics and opioids are frequently used following traumatic brain injury, but cannot be used long-term. Both of these pharmacological agents could delay the immediate identification of PSH. It is plausible that other hormonal disturbances associated to the injury may also appear by the time of PSH identification following the discontinuation of anesthetics and opioids. The hormonal panel could serve the purpose of establishing comparative values for identifying future disturbances in pituitary hormones after injury onset. Initiating appropriate hormonal management parallel to the management for PSH, may optimize patient outcomes.

There is no consensus about the incidence of PSH, with rates being reported between 6 and 33% (25–27). This has been hypothesized to result from the lack of recognition of PSH. While the frequency of PSH following TBI appears to be higher (closer to the 33%), other conditions such as the ones presented herein have an estimated prevalence of 6% (12). In all the encounters evaluated for this report, only one patient had a documented diagnosis of PSH, revealing the lack of recognition or effective documentation. The reasons for the lack of recognition are multifactorial. There is no unique ICD code for the documentation of PSH. As we report in the methods, ICD codes given to our patients were not specific for the PSH diagnosis. It is only from an in-depth assessment of the patients' documented symptoms, laboratory values, and medications that we were able to pinpoint the current cohort of nine patients. In addition, there might be a significant lack of recognition of PSH by medical personnel (28). Increased education to critical care nurses (15), and doctors involved in the treatment of patients at risk of developing PSH should prove effective in regards to identification and treatment. Early identification and treatment could lead to reduced morbidity and mortality (28).

The severity and the presence of multiple symptoms associated with the autonomic system over activity should be an alert to examine a patient using the PSH diagnostic likelihood scale. While the applicability and value of the scale were validated in the current dataset, we found it to be complicated. Applying and using the scale bedside could be cumbersome as multiple criteria need to be recorded. However, collecting the observations of the patient bedside represents an increased benefit, since retrospective calculations (chart review) face the risk of missing data and/or inaccurate documentation. For example, if a patient presents with rigidity or spasticity (although not always present), this should trigger an alert for the assessment of PSH, moreover if there's a history of TBI. While GCS and ICP are not criteria included in the assessment of PSH, we found that it could serve as additional indicators of the patient's overall condition and progress during PSH.

Previous research has discussed the inefficacy of monotherapy for the management of PSH (15). The main indication of opioids is for pain management, but it also helps in controlling hypertension and tachycardia in patients with PSH. On the contrary, the treatment of hypertension may require multiple agents, such as beta-blockers and calcium channel blockers. It is noticeable that patients were primarily maintained on the following pharmacological classes (after discharge) to control PSH: beta-blockers, opioids, and neuromodulators, plus the thyroid hormone. Due to the design of the study, we were not able to assess the long-term impact of the medications on symptom management post-discharge.

### Clinical Recommendations

Based on the current observations from our cohort of Hispanic patients, we present a comparative assessment in order of preference of use for controlling refractory symptoms of PSH. Anesthetics and opioids emerged as the top agents to use. Propofol was found to have a better effect than dexmedetomidine. Propofol is a short-acting intravenous anesthetic that can provide sedation within 40 s. This rapid anesthesia, with inhibition of respiratory and circulatory systems are believed to assist in controlling PSH symptoms such as hypertension, tachycardia, and tachypnea. Opioids also helped control most of the refractory symptoms in the following order of preference: fentanyl (IV push, IV drip, or patch), morphine, and lastly, meperidine. It is hypothesized that opioids worked well possibly by producing a dual effect: decreasing the natural fight or flight response to the pain (reduction of pain), thus blunting sympathetic activity or via a direct effect of opioid receptors in other areas of the brain such as the hypothalamus (29). It appears that it is easier to control blood pressure (BP) and tachycardia when opioid analgesics, such as fentanyl, are given concomitantly. Generally, beta-blockers helped in controlling HR and BP. The calcium channel blocker, nicardipine, lowered BP in all three of the trauma patients but had no effect on tachycardia. We postulate that this could be the drug of choice as an agent to acutely decrease BP. Hydralazine showed no effect in controlling PSH symptoms. Hypertension and respiratory rate were controlled adequately with benzodiazepines. Finally, neuromodulators were shown to have some effect in controlling spasticity. However, the full spectrum of activity of neuromodulators remains unanswered since the clinical evaluation of the patient's response was limited in the medical records.

### Limitation of the Study

There were strict inclusion and exclusion criteria for this study. This implies that despite applying the rigorous verification process of the charts, we might have missed a possible undiagnosed case of PSH. Nevertheless, this highlights the urgent need for a coding system that effectively identifies this group of patients. As in any retrospective study, we depend upon the quality of the data entered by clinical personnel. For example, the assessment of sweating was challenging as only the presence or absence of it was noted in the charts and rarely the severity of it. The cohort of patients in this study was all of the Hispanic ethnicity, which represents the population of our region, but places the limitation of generalizing the results to other populations. Future studies should consider long term outcomes, and consider whether early symptom control provides increased benefits to the patients.

## Conclusion

Due to the lack of knowledge of PSH, it is strongly recommended to create an ICD code description for easier identification. Moreover, training to nurses and other medical personnel in charge of caring for patients with brain insults should be augmented. Medication management did not follow a specific pattern, suggesting that more studies need to focus on this area. The critical finding presented here regarding the delayed initiation of thyroid hormone replacement suggests that the pituitary axis might have a role in the pathophysiology of PSH. Additional technology could assist in a better understanding of pathways that explain PSH origin.

## Data Availability Statement

The authors did not have permission from the IRB to deposit the data used in this article in a public repository. Raw data can be shared with interested individuals by contacting the corresponding author.

## Ethics Statement

The studies involving human participants were reviewed and approved by DHR Health Institute for Research and Development IRB. Written informed consent from the participants' legal guardian/next of kin was not required to participate in this study in accordance with the national legislation and the institutional requirements.

## Author Contributions

All authors contributed to the literature extraction and prepared the manuscript. AKA extracted and analyzed the data. ATR synthesized the data and ran statistical analyses. JMP supervised the study. All authors read and approved the study.

## Conflict of Interest

The authors declare that the research was conducted in the absence of any commercial or financial relationships that could be construed as a potential conflict of interest.
